# Multi-omics dissection of R-loop dynamics in tumorigenesis: From transcription-replication conflict to therapeutic targets

**DOI:** 10.1016/j.omton.2026.201237

**Published:** 2026-05-14

**Authors:** Yu Zhang, Nuorong Xiong, Yueyang Song, Xiaojie Lu, Kai Ni, Zijian Tang

**Affiliations:** 1 College of Biomedicine and Health, Huazhong Agricultural University, Wuhan, Hubei 430070, China; 2 Department of General Surgery, Liver Transplantation Center, The First Affiliated Hospital of Nanjing Medical University, Nanjing, Jiangsu 210029, China; 3 Department of Urology, Shanghai Sixth People’s Hospital Affiliated to Shanghai Jiao Tong University School of Medicine, Shanghai 200233, China

**Keywords:** genomic instability, multi-omics, R-loops, therapeutic targets, transcription-replication conflicts

## Abstract

R-loops, three-stranded nucleic acid structures comprising an RNA-DNA hybrid and a displaced single-stranded DNA strand, play context-dependent roles in cancer—serving essential physiological functions while also driving tumorigenesis when dysregulated. Their pathological effects are mediated through replication stress, genomic instability, transcriptional disruption, and defective RNA processing. This review highlights the emerging potential of targeting R-loops as a therapeutic strategy in oncology. We survey advanced methodologies for R-loop mapping, addressing technical limitations of current approaches, and advocate for multi-omics integration to elucidate R-loop dynamics and functional networks in cancers such as lung, bladder, and prostate malignancies. Critically, we explore two promising therapeutic avenues: (1) direct inhibition of R-loop resolvers to trigger excessive R-loop accumulation and replication catastrophe, and (2) synthetic lethality strategies that capitalize on cancer-specific R-loop handling defects. Clinical evidence supporting these approaches is discussed, along with challenges including tumor heterogeneity, detection limitations, and adaptive resistance. We argue that a multi-omics-driven understanding of R-loop biology will accelerate the translation of R-loop-directed therapies into precision oncology.

## Graphical abstract

**Figure:**
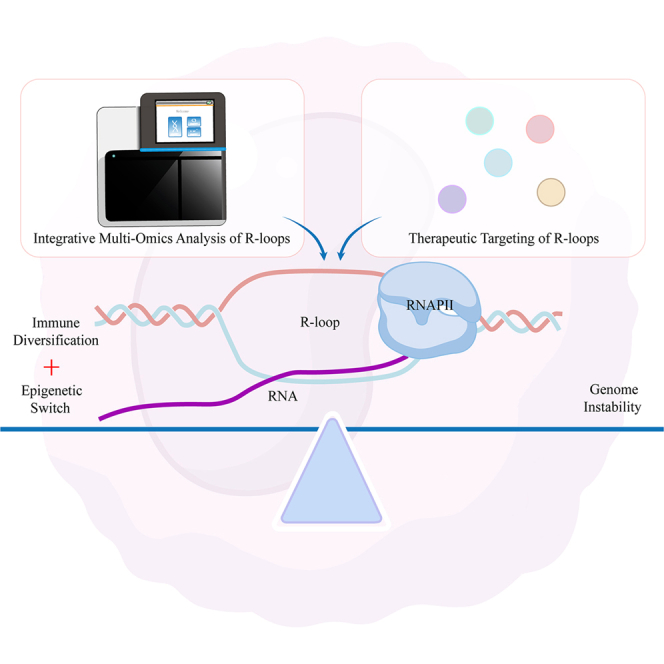

## Teaser

R-loops are double-edged players in cancer—essential for cellular function yet drivers of malignancy when dysregulated. This review highlights their therapeutic potential, focusing on direct inhibition and synthetic lethality strategies, and argues that multi-omics integration is key to advancing R-loop-directed precision oncology.

## Introduction

**Figure 1:**
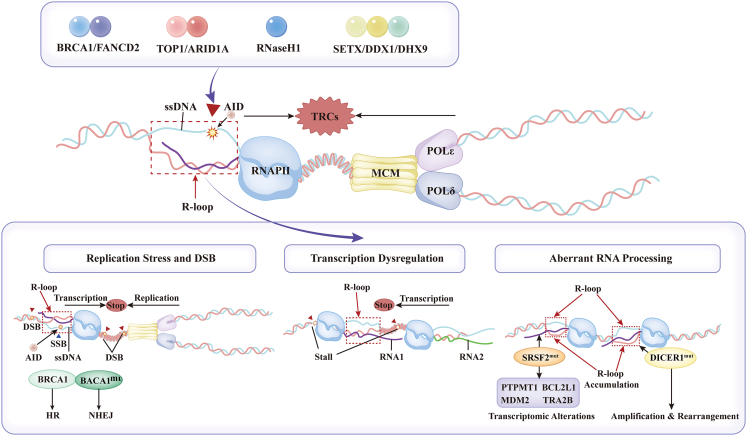
Molecular mechanisms connecting R-loops with replication stress and DSB, transcription dysregulation, and aberrant RNA processing R-loops are triple-helical nucleic acid structures comprising an RNA:DNA hybrid duplex and a displaced single-stranded DNA molecule. These structures pose dual threats to genomic stability: they stall replication forks by physically obstructing helicase progression and immobilize RNA polymerase II, triggering DNA damage responses and transcriptional arrest. To prevent genomic catastrophe, cellular safeguard mechanisms actively resolve pathological R-loops. Failure of these controls propagates DNA damage, fueling tumor evolution. Key resolution enzymes (including BRCA1/FANCD2, TOP1/ARID1A, RNaseH1, and SETX/DDX1/DHX9) protect genomic integrity; notably, exposed ssDNA can activate and be cleaved by AID. Furthermore, R-loops triggered by TRCs can directly mediate double-strand breaks and single-strand breaks (SSBs). Mutations impairing BRCA1 function weaken homologous recombination (HR), favoring error-prone non-homologous end joining (NHEJ). Persistent R-loops also cause transcriptional dysregulation and promote transcription-associated DSBs. Additionally, mutations in splicing factors like SRSF2 induce RNAPII stalling and increase R-loop formation, leading to aberrant splicing of genes such as PTPMT1, BCL2L1, MDM2, TRA2B, and MKNK2, which alters transcriptional function. Finally, R-loop accumulation driven by DICER1 mutations manifests as gene amplification and chromosomal rearrangements.

The R-loops represent a triple-helical nucleic acid structure characterized by an RNA: DNA hybrid duplex and a displaced single-stranded DNA (ssDNA) molecule.^1^ Its formation is intrinsically coupled to transcriptional dynamics: nascent RNA anneals to the template DNA strand within the transcription bubble, driven mechanistically by elevated transcriptional flux, negative supercoiling accumulation, and GC-skewed genomic sequences that stabilize RNA:DNA hybrids.^2^^,^^3^ This triplex conformation is synergistically maintained through topoisomerase-mediated resolution of torsional stress and chromatin-modifying complexes that regulate DNA accessibility (Figure 1).^4^

Crucially, R-loops function as essential physiological regulators rather than genomic instability artifacts.^5^ In physiological settings, R-loops fulfill essential cellular functions, particularly in immunoglobulin class—switch recombination and the regulation of epigenetic states.^6^^,^^7^^,^^8^^,^^9^^,^^10^ However, dysregulation of their homeostasis directly contributes to DNA damage and disease pathogenesis, highlighting their duality in cellular physiology.^11^^,^^12^ Thus, whether an R-loop is considered functional or pathological is entirely context-dependent, determined by the balance between its beneficial and detrimental effects.^13^ Importantly, these two categories are not mutually exclusive and can undergo dynamic transitions under specific cellular conditions.^14^ For example, in some contexts, R-loops are clearly functional: they promote H3K79 methylation, interact with the cohesin complex, and contribute to the establishment of three-dimensional (3D) chromatin architecture.^15^ In others, however, they disrupt chromatin homeostasis and become pathological.1^16^ This context-dependent switch underscores the delicate equilibrium that governs R-loop functionality and highlights how the same molecular entity can exert opposing effects depending on cellular state and genomic context.

**Figure 2:**
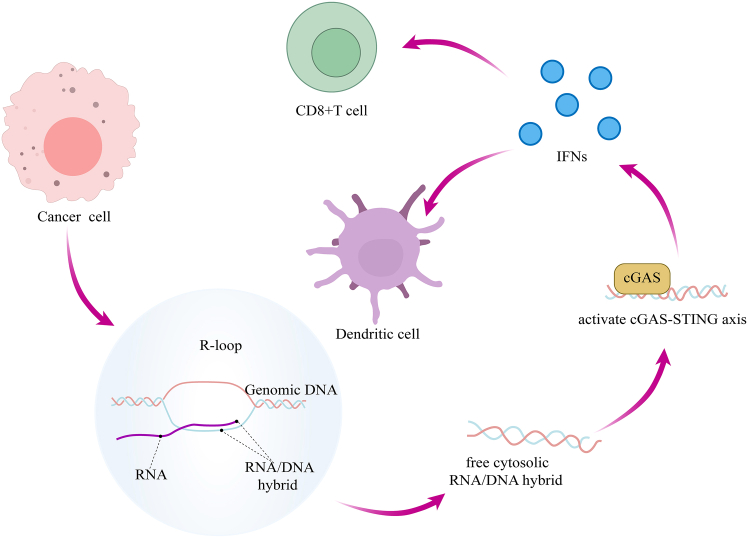
R-loops trigger antitumor immunity through activation of the cGAS-STING pathway The antitumor immune response initiated by R-loops unfolds in three key stages: first, R-loops accumulate in the nucleus and release RNA-DNA hybrids into the cytosol. Second, these hybrids are sensed by cGAS, activating the cGAS-STING-IFN signaling axis. Finally, the resulting interferon response stimulates dendritic cells and CD8^+^ T cells, culminating in potent antitumor immunity.

This functional duality extends to oncogenesis, where R-loops exhibit pro- or anti-tumorigenic roles dictated by genomic context.^17^ This pathogenic potential exerts context-dependent pro-tumorigenic effects, such as driving oncogene expression (e.g., *MYC*, *E2F3*) and exacerbating homologous recombination (HR) deficiency, thereby fueling chromosomal aberrations.^18^^,^^19^^,^^20^^,^^21^^,^^22^^,^^23^^,^^24^^,^^25^^,^^26^ Conversely, R-loops also exhibit context-dependent anti-tumorigenic properties, including activating innate immune responses.^27^^,^^28^ For instance, the rapid proliferation of tumor cells leads to the accumulation of excessive R-loops within the nucleus, triggering cytoplasmic immune sensing (cGAS) and ultimately activating systemic anti-tumor immunity (Figure 2).

**Figure 3:**
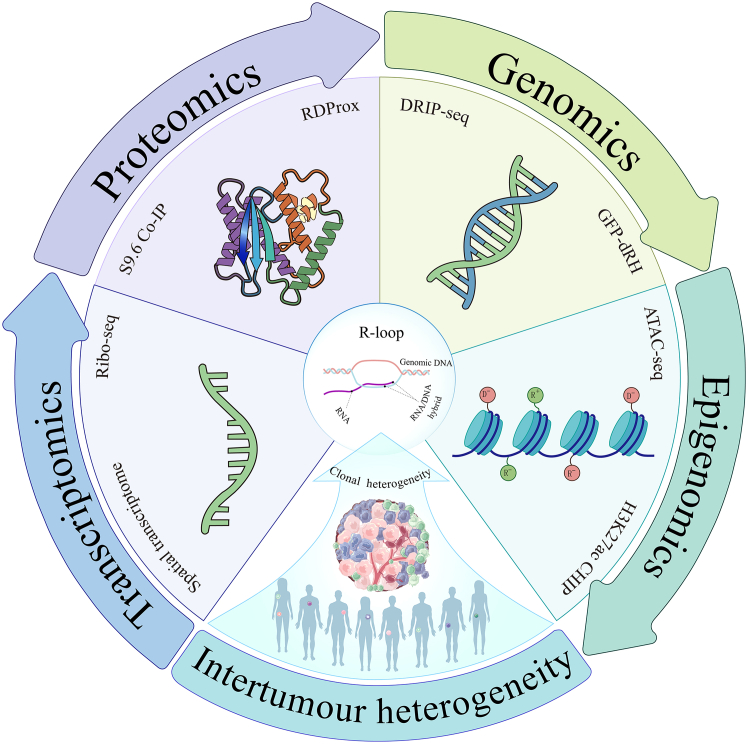
Multi-omics integration strategy for detecting R-loops Tumor heterogeneity necessitates spatially resolved multi-omics to dissect R-loop dynamics. First, integrating DRIP-seq with GFP-dRH live-cell imaging pinpoints R-loop genomic locations. Second, combined ATAC-seq/H3K27ac-seq profiling reveals R-loop co-occupancy with open chromatin and active enhancers, linking them to epigenetic regulation. Third, spatial transcriptome and Ribo-seq integration builds a multi-layered model from transcription initiation through mRNA distribution to translational output, comprehensively defining R-loop roles within gene networks. This uncovers novel biology obscured in single-dimensional analyses and establishes R-loop-mediated transcription-translation coupling. Finally, S9.6 CoIP/RDProx combined with IP-MS identifies R-loop-associated proteins and interaction networks, yielding actionable therapeutic targets.

However, the successful translation of these therapeutic strategies hinges on a deeper understanding of R-loop dynamics—a goal that remains constrained by current methodological limitations. Existing approaches, predominantly antibody-dependent or nuclease-based, enable genome-wide mapping but suffer from critical shortcomings such as dynamic blindness (failure to capture real-time R-loop—epigenetic modifier interactions) and spatial resolution deficits (inability to resolve context-dependent heterogeneity across genomic compartments or 3D architectures).^29^^,^^30^^,^^31^^,^^32^^,^^33^^,^^34^ To overcome these limitations, an integrated multi-omics framework must unify transcriptomic dynamics to define functional couplings between R-loop kinetics and transcriptional bursting, proteomic profiling to capture R-loop-interacting protein complexes, genomic mapping to resolve sequence determinants of R-loop accumulation, and epigenomic interrogation to decode dynamic cross talk among chromatin modifiers (Figure 3).^35^^,^^36^^,^^37^^,^^38^ Such integration is essential to dissect the spatiotemporal governance of R-loop functions in modulating transcriptional plasticity, epigenetic memory, and genomic stability.

This review bridges fundamental nucleic acid biology with clinical oncology by centering on three interconnected themes: the mechanistic machinery governing R-loop homeostasis, the integrative multi-omics approaches for profiling R-loop dynamics, and the emerging therapeutic strategies targeting R-loop vulnerabilities in cancer. By integrating mechanistic understanding with technological advances, this review provides a framework for translating R-loop biology into clinically actionable strategies.

### Molecular mechanisms of R-loop-driven tumorigenesis

Transcription-replication conflicts (TRCs) arise from the spatial and temporal competition between replication forks and transcription complexes for genomic DNA templates.^39^ While co-directional TRCs are generally resolved with minimal genomic instability, head-on conflicts (characterized by antagonistic movement of replisomes and replication fork progression and trap RNA polymerase II (RNAPII) promote pathological R-loop accumulation.^40^^,^^41^ These structures physically block RNAPII, triggering replication stress, DNA damage responses, and transcriptional arrest.^42^ Failure to resolve such conflicts compromises genome stability and creates a permissive environment for oncogenic transformation.^43^

To counteract such threats, cells deploy specialized enzymatic regulators that mitigate R-loop accumulation through two primary strategies—alleviating DNA torsional stress and destabilizing RNA:DNA hybrids. Among these, the representative DNA topoisomerase I (TOP I) resolving conflicts at transcription-replication interfaces through transient ssDNA cleavage and re-ligation cycles to dissipate torsional strain.^44^ Additionally, the ARID1A chromatin remodeler recognizing R-loops via ATM-dependent phosphorylation to recruit the METTL3/14 complex for m^6^A methylation on the RNA moiety, an epitranscriptomic mark enabling RNase H1-dependent degradation.^45^ A further major class of ATP-dependent RNA helicases (e.g., SETX, DDX1, DEAD-box helicase 5 [DDX5], and DHX9) dissociating hybrids via strand displacement. Moreover, DNA damage sensors (BRCA1 and FANCD2) localizing to R-loop sites to recruit HR factors, collectively preserving genomic integrity.^46^^,^^47^^,^^48^

When these protective systems fail, R-loops become potent drivers of oncogenesis. In the following sections, we dissect three core pathological axes: (1) R-loop-induced replication stress and genome instability, (2) R-loop-driven transcriptional dysregulation, and (3) cross talk between R-loops, aberrant splicing, and microRNA (miRNA) processing defects in cancer.

### R-loop-induced replication stress and genome instability

R-loop formation elevates local DNA super helicity and imposes severe torsional stress on replication forks. Persistent R-loops arising from TRCs can lead to replication fork stalling or collapse.^49^ Inadequate resolution of this stress by TOP I induces DNA structural distortions or, under persistent tension, double-strand break (DSB) formation.^50^^,^^51^ These lesions activate DNA damage sensor kinases (e.g., ATM/ATR) and initiate repair machineries.^52^ To mitigate genomic instability, cells engage immediate DNA repair responses; however, in the neoplastic microenvironment, concomitant deficiencies in R-loop regulatory factors and DNA repair components result in pathological R-loop accumulation, driving profound genomic catastrophe characterized by elevated DSB burden.^53^ Accumulated R-loops may also mask DNA damage sites, interfering with key steps in transcription-coupled repair—including nucleotide excision repair and HR—thereby exacerbating repair defects and facilitating mutation accrual.^54^ Such instability promotes carcinogenesis through localized copy number alterations, including tumor suppressor gene deletions and oncogene amplifications.^14^^,^^32^

Persistent R-loops also drive chromosomal rearrangements, as observed in leukemia. For example, circMLL RNA forms a circular R-loop (circR-loop) with its cognate DNA, recruiting a proteasome-inhibitory complex that induces RNAPII stalling, DSBs, and oncogenic translocations, thereby accelerating leukemogenesis *in vivo*.^55^ Additionally, spliceosome-mutant leukemias exhibit R-loop-driven mitotic DNA damage and chromosomal mis-segregation, further amplifying genomic instability.^56^ Critically, R-loops establish a self-reinforcing pathogenic circuit: initial mutations induce HR deficiency, impeding DSB repair and accelerating mutagenesis. For instance, cyclin E overexpression exacerbates TRCs, causing replication fork collapse and DNA damage that fosters oncogenic mutations.^57^ Similarly, oncogenic HRas^V12C^ upregulates TATA binding protein (TBP), driving transcriptional hyperactivity; concomitant R-loops stall replication forks, and induce DNA breakage.^58^ Together, these events convert normal genome surveillance into a pathogenic engine driving tumorigenesis.

### R-loop-driven transcriptional dysregulation

Unscheduled R-loops induce transcription stress, characterized by excessive pausing, arrest, or backtracking of RNA polymerases (RNAPs)—a process tightly interconnected with R-loop formation, as persistent RNAP stalling itself elevates local R-loop levels.^59^^,^^60^ When deregulated R-loops cause R-loop-mediated transcription stress, the nucleotide excision repair endonucleases XPG and XPF can cleave DNA, creating a single-stranded gap that often progresses into DSBs, triggering downstream DNA damage repair pathways in both cycling and non-cycling cells.^61^

In liver cancer, GPATCH4 recruits DDX21 to clear R-loops from the nucleolus, boosting ribosomal RNA (rRNA) transcription and tumor proliferation.^62^ In glioblastoma, R-loop buildup at loci like MYC and E2F3 increases oncogene expression to drive carcinogenesis.^20^ Intriguingly, in ovarian cancer, R-loop accumulation within the *BRCA1* promoter region hinders recruitment of DNA repair machinery, leading to *BRCA1* promoter hypermethylation and transcriptional silencing.^63^ These examples illustrate how R-loop dysregulation directly contributes to oncogenic transcriptional programs.

### R-loops converge with aberrant RNA processing

R-loops arising from TRCs disrupt post-transcriptional processing networks by impairing RNAPII processivity and compromising spliceosome assembly through disrupted splicing factor recruitment or splice site recognition.^64^^,^^65^^,^^66^ This paradigm is exemplified in Wiskott-Aldrich syndrome (WAS), where deficiency of the WAS protein (WASp) induces pathological R-loop accumulation and spliceosome disassembly at specific genomic loci, resulting in aberrant transcript processing.^67^^,^^68^

R-loop-associated splicing dysregulation contributes directly to oncogenesis, as evidenced in myelodysplastic syndromes (MDS)—clonal hematopoietic malignancies characterized by ineffective hematopoiesis, morphological dysplasia, and heightened progression risk to acute myeloid leukemia (AML).^69^ Somatic mutations in spliceosome components (e.g., *SF3B1*, *U2AF1/U2AF35*, *SRSF2*, and *ZRSR2*) are prevalent in MDS. Mechanistic studies reveal that mutant SRSF2 disrupts *p*-TEFb translocation from the 7SK small nuclear ribonucleoprotein particle (snRNP) complex to RNAPII, inducing transcriptional elongation defects that potentiate R-loop elevation—thereby establishing a direct etiological link between spliceosome dysfunction and R-loop-mediated genomic instability.

Beyond splicing, R-loops interfere with miRNA biogenesis by forming near the transcription start site region of miRNA genes, and promote co-transcriptional pri-miRNA processing.^70^ This disruption of miRNA homeostasis potentiates chromosomal break points and genomic rearrangements during tumor progression. A paradigm of this oncogenic mechanism occurs in embryonal tumors with multilayered rosettes (ETMRs)—highly aggressive pediatric central nervous system (CNS) malignancies encompassing embryonal tumor with abundant neuropil and true rosettes (ETANTR), ependymoblastoma, and medulloepithelioma.^71^ The proposed driver of ETMRs involves chromosome 19q13.42 abnormalities at the *C19MC* miRNA cluster locus, manifesting as either focal amplification or oncogenic *TTYH1:: C19MC* fusions, which drive overexpression of *C19MC* miRNAs and an embryonic brain-specific *DNMT3B* isoform.^72^^,^^73^ Pathological R-loop accumulation at this locus induces chromosomal instability and breakage, directly facilitating *C19MC* amplification/rearrangement in ETMRs. Intriguingly, a subset of ETMRs harbors damaging *DICER1* mutations clustered in its helicase and RNase III domains (somatic or germline). DICER1 deficiency—a known cancer predisposition lesion—compromises processing of *C19MC*—transcribed miRNAs, thereby establishing a synergistic axis, wherein R-loop-mediated DNA damage and miRNA processing failure cooperatively potentiate tumorigenesis.^74^

Collectively, dysregulated R-loops drive oncogenesis through a self-reinforcing cycle of genomic instability, transcriptional dysregulation, and magnify aberrant RNA splicing and miRNA cleavage. These paradigms positions R-loops not merely as byproducts of TRCs but as central mediators that initiate and sustain malignant transformation.

### Multi-omics technologies for dissecting R-Loop dynamics

Diverse high-throughput sequencing methodologies facilitate genome-wide mapping of R-loop distribution across various cell types and genomic regions.^29^^,^^75^ These techniques predominantly employ two distinct detection principles: immunoprecipitation utilizing the S9.6 monoclonal antibody specific for RNA-DNA hybrids, or affinity enrichment via catalytically inactive RNase H (dRNase H) enzymes.^5^ Each strategy exhibits characteristic advantages and limitations. S9.6 antibody-based detection leverages the direct correlation between antibody binding affinity and RNA-DNA hybrid abundance, enabling quantitative assessment of R-loop levels.^76^ Signal intensity thus serves as a proxy for relative R-loop density. S9.6-based methods offer intuitive data interpretation and have been widely implemented for high-resolution (base-pair level) genome-wide mapping.^77^ However, they are subject to significant limitations: (1) Potential cross-reactivity with non-canonical nucleic acid structures (e.g., G-quadruplexes [G4] and RNA triplexes) necessitates stringent RNase H pretreatment controls to mitigate false positives and (2) Steric hindrance within highly condensed chromatin domains (e.g., heterochromatin) can impede antibody accessibility, leading to systematic underestimation of R-loop signals in these regions.^78^ In contrast, catalytically inactive RNase H (dRNase H)-based detection enables direct spatial mapping of R-loops through localization of the dRNase H binding site, typically visualized via fluorescence colocalization.^79^ It is particularly advantageous for subcellular-resolution mapping and facilitates analyses of R-loop association with specific nuclear compartments (e.g., nucleoli). Analogous to chromatin immunoprecipitation sequencing (ChIP-seq), sequencing of dRNase H-bound genomic regions (e.g., dRH sequencing) can generate genome-wide R-loop profiles.^30^^,^^80^ This method potentially offers enhanced specificity and reduced background noise compared to antibody-based approaches. However, key limitations include: (1) The requirement for intracellular delivery (e.g., transfection and viral transduction) introduces potential artifacts, as dRNase H overexpression may competitively bind R-loops and disrupt their native dynamics and physiological functions; (2) dRNase H typically exhibits lower binding affinity for RNA-DNA hybrids compared to the S9.6 antibody, thereby limiting sensitivity for low-abundance R-loops.^81^ Given these complementary strengths and weaknesses of the two primary approaches, their combined application is strongly recommended to enhance experimental robustness and data reliability. This may involve utilizing dRNase H-based mapping to validate S9.6 antibody specificity or integrating dRNase H binding profiles with S9.6-derived genome-wide data for calibration. Building on the foundation of these core principles (S9.6 antibody or dRNase H), numerous high-throughput methods have been developed for R-loop detection. We will now detail several key techniques, categorized by their primary detection mechanism.

### S9.6 antibody-based R-loop mapping techniques

DRIP-seq (DNA:RNA hybrid immunoprecipitation and sequencing) represents the predominant high-throughput sequencing method for *in vitro* R-loop enrichment, achieving genome-wide coverage of R-loop distributions.^29^^,^^75^^,^^82^ This methodology initiates with nucleic acid extraction from unfixed fresh cells to preserve native RNA-DNA hybrid conformations.^83^ Following restriction endonuclease digestion (e.g., HindIII, EcoRI, and BstXI) generating 5–10 kb genomic fragments, *in vitro* R-loop enrichment is performed using the S9.6 monoclonal antibody specific for RNA-DNA hybrids.^77^^,^^78^ Subsequently, immunoprecipitated DNA strands are subjected to high-throughput sequencing, thus permitting genome-wide profiling of R-loop distribution patterns.^31^ Through integrated applications in high-throughput drug screening, transgenic murine models, and clinical tumor specimen analyses, DRIP-seq has systematically delineated associations between R-loop dysregulation and therapeutic resistance.^84^ However, DRIP-seq exhibits two primary technical limitations: (1) cross-reactivity artifacts arising from antibody affinity for double-stranded RNA, which generates false-positive signals; and (2) resolution constraints due to kilobase-scale genomic fragments produced by restriction endonuclease digestion, resulting in broad R-loop signal regions.^85^ To circumvent the technical bottlenecks associated with conventional DRIP-seq, several enhanced variants have emerged: (1) DRIPc-seq (DNA-RNA immunoprecipitation followed by cDNA conversion coupled to sequencing) mitigates antibody cross-reactivity through cDNA library construction from immunoprecipitated RNA strands, enabling strand-of-origin resolution and transcriptional directionality mapping—an approach pivotal for elucidating estrogen-induced R-loop dynamics in breast cancer models2^29^^,^^86^; (2) S1 nuclease (S1)-DRIP-seq achieves precise localization by replacing restriction digestion with sonication fragmentation coupled with S1 nuclease-mediated ssDNA elimination, revealing 781 transcription-associated hybrid-sensitive regions beyond canonical sites8^87^; (3) ssDRIP-seq (ssDNA ligation-based library construction of DRIP-seq) employs strand-specific adapter ligation (Adp1/Adp2 to 3′/5′ ssDNA ends) to preserve strand information while circumventing reverse transcription bias9^9^^,^^88^; (4) ULI-ssDRIP-seq (ultra-low input ssDNA ligation-based library construction of DRIP-seq) extends this framework to near single-nucleotide resolution, facilitating discoveries such as R-loop enrichment at enhancers during zebrafish zygotic genome activation (ZGA) regulating sox3/has2 expression8^89^; (5) R-loop CUT&Tag (R-loop cleavage under targets and tagmentation) overcomes *in vitro* artifacts by deploying Tn5 transposase for *in situ* R-loop cleavage, thereby enabling direct correlation of R-loops with epigenetic modifications and DNA repair pathways.^90^^,^^91^ Collectively, these S9.6 antibody-based methodologies provide critical frameworks for genome-wide R-loop localization and orientation analysis9^92^; and (6) S9.6 co-IP (immunoprecipitation) coupled proteomics characterizes proteins that bind R-loops to control their homeostasis or mediate downstream activities.^36^

### dRNase H-based R-loop mapping techniques

To overcome limitations inherent in S9.6 antibody-dependent *in vitro* enrichment, endogenous R-loop capture methodologies leveraging dRNase H have emerged as a critical research frontier. Early efforts include DRIVE-seq (DNA:RNA *in vitro* enrichment and sequencing), which utilizes dRNase H for RNA-DNA hybrid capture. Despite limited sensitivity (detecting only 1,224 peaks versus 20,862 in S9.6-based methods), it established a foundational framework for subsequent development.^93^ Although DRIVE-seq demonstrated limited sensitivity, its pioneering use of dRNase H established a critical paradigm for endogenous R-loop capture. Building upon this foundation, subsequent innovations significantly enhanced detection efficacy and scope: (1) R-ChIP (R loop chromatin immunoprecipitation) leverages stably transfected cell lines expressing RNase H1 catalytic domain mutants (e.g., D210N) to achieve *in situ* enrichment, enabling the elucidation of pathological linkages between splicing factor mutations, R-loop accumulation, and genomic instability in MDSs3^30^^,^^67^^,^^94^; (2) MapR integrates R-ChIP with CUT&Tag methodology through exogenous micrococcal nuclease (MNase)-mediated *in situ* chromatin release, enhancing sensitivity >5-fold while reducing background noise for robust genome-wide R-loop locus identification9^95^^,^^96^; (3) RIAN-seq (R-loop identification assisted by nuclease and sequencing) deploys a triple nuclease system (nuclease P1/T5 exonuclease/λ exonuclease) to resolve dynamic R-loop regulation across physiological-pathological spectra, thereby identifying evolutionarily conserved DNA damage-susceptible R-loops (DdsR-loops) as drivers of eukaryotic genomic instability9^97^; (4) GFP-dRH live-cell tracing utilizes catalytically inactive RNase H1 (dRH)-GFP fusions to permit real-time subcellular tracking of R-loop spatiotemporal dynamics via confocal microscopy, particularly enabling analysis of low-input specimens including early embryos and stem cells7^79^; and RDProx (R-loop-proximal protein networks using RNA-DNA proximity proteomics) facilitates the mapping of the R-loop-proximal proteome through the use of a fusion protein comprising the hybrid-binding domain (HBD) of RNaseH1 and an engineered version of ascorbate peroxidase (APEX2).^35^ While current dRNase H-based methods provide comprehensive genome-wide R-loop landscapes, they remain constrained to static snapshots. Future innovations must further dissect R-loop spatiotemporal dynamics to fully unravel their regulatory roles in cellular physiology and disease pathogenesis.

### Multi-omics integration strategies for R-loop dynamics

Beyond mapping techniques, integrating R-loop data with other molecular layers through multi-omics approaches has proven powerful for uncovering their functional roles, especially in disease contexts like cancer. This strategy establishes a systematic framework for resolving R-loop-enriched genomic regions and their interplay with DNA repair mechanisms through integrative analysis of genomic, transcriptomic, and proteomic data.^31^^,^^98^ For instance, in lung cancer, combinatorial whole-transcriptome microarrays, liquid chromatography-tandem mass spectrometry (LC-MS/MS), and R-ChIP revealed that p53 suppresses DNA damage-induced R-loop accumulation and enhances chemoresistance by activating ASCC3 transcription, facilitating circASCC3 biogenesis and its interaction with DDX5.^99^ In bladder cancer, convergent RNA sequencing (RNA-seq), DRIP-seq, and LC-MS/MS analyses demonstrated that NOP2/Sun RNA methyltransferase 2 (NSUN2) catalyzes m^5^C-mediated R-loop modification, synergizing with zeste homolog 2 (EZH2) to epigenetically silence PRDM11 transcription.^100^ In prostate cancer, multi-platform profiling (LC-MS/MS, DRIP-seq, m^6^A DIP, RNA-seq, and MethylationEPIC BeadChip) identified insulin-like growth factor 2 mRNA-binding proteins (IGF2BPs) as amplifiers of R-loop signaling across promoter regions—notably at the SEMA3F locus. Mechanistically, IGF2BPs drive oncogenic SEMA3F overexpression by evicting DNMT1 and YTHDF2 methylreaders.^101^ Collectively, these cross-cancer investigations establish multi-omics as a transformative paradigm for deciphering R-loop-driven oncogenic circuits. By integrating maps of R-loop topography with epigenetic modifiers and transcriptional outputs, this approach reveals targetable vulnerabilities that link R-loop dysregulation to tumor evolution.

### Challenges and future directions for multi-omic detection of R-loop dynamics

The advent of multi-omics has brought unprecedented opportunities for deciphering R-loop dynamics. However, harnessing its full potential for precise and comprehensive R-loop monitoring requires overcoming two core challenges.

One major hurdle lies in technological limitations of current detection methods. Current R-loop detection methodologies predominantly rely on S9.6 antibody-based immunoprecipitation or RNase H1-dependent enzymatic approaches. While enabling genome-wide mapping, these techniques exhibit critical limitations: insufficient spatiotemporal resolution, unresolved probe specificity controversies, limited dynamic tracking capability, and inadequate resolution of tumor heterogeneity. Consequently, the development of single-molecule fluorescence hybridization probes represents a strategic priority for advanced R-loop detection.^80^ Ideal probes must simultaneously satisfy three critical criteria: (1) high specificity for RNA-DNA hybrids with minimal cross-reactivity to dsDNA or ssRNA; (2) structural adaptability to diverse R-loop conformations; and (3) sensitivity sufficient for low-abundance targets.

Compounding these technological challenges is the computational complexity of integrating multi-omics data. The dimensionality discordance between epigenetic and R-loop profiles, coupled with the difficulty in distinguishing causal from correlative interactions, requires advanced algorithmic solutions. Overcoming these barriers necessitates the development of novel artificial intelligence (AI) technologies. Pioneering frameworks such as scDART (single-cell multi-omics integration tool), a deep learning model integrating scRNA-seq (single-cell RNA sequencing) and scATAC sequencing data to simultaneously learn cross-modality relationships, preserve cell trajectories within continuous populations, and enable trajectory inference on integrated data.^102^ Similarly, DeepER, a deep learning-enhanced R-loop prediction tool, demonstrates superior performance in genome-wide R-loop annotation, providing deeper insights into the position- and context-dependent effects of nucleotide composition on R-loop formation. DeepER also reveals a strong association between specific tandem repeats and R-loop formation, paving the way for understanding the mechanisms underlying certain repeat expansion diseases.^103^

In summary, the continued advancement of multi-omics technologies holds great promise for unraveling the complex regulatory networks that govern R-loop dynamics. Integrating genomic, transcriptomic, and epigenomic dimensions will ultimately illuminate how these structures shape genome architecture and function in health and disease.

### R-loops as therapeutic targets

**Figure 4:**
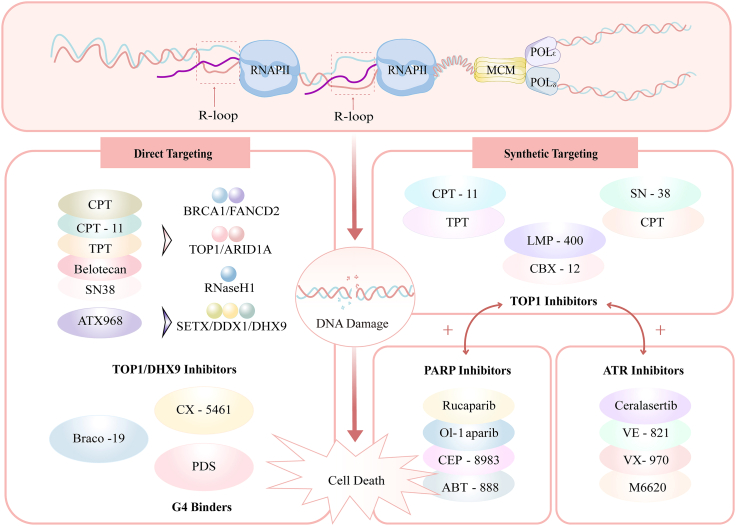
R-Loops as emerging therapeutic targets in cancer R-loop-targeted anticancer strategies operate through two core mechanisms: left: directly inhibiting key regulators to drive harmful R-loop buildup and replication failure. This includes TOP1/2 inhibitors (CPT, topotecan, belotecan, irinotecan, and its active metabolite SN38); DHX9 inhibitor (ATX-968); and G4 ligands (CX-5461, PDS, Braco-19). Right: taking advantage of synthetic lethality to worsen existing R-loop defects in cancer cells. Examples include combining PARP inhibitors (rucaparib, olaparib, CEP-8983, ABT-888), or ATR inhibitors (ceralasertib, VE-821, VX-970) with TOP1 inhibitors (LMP-400, CBX-12).

Dysfunction or mutation of R-loop regulatory factors compromises R-loop homeostasis and creates actionable vulnerabilities in cancer cells. Pharmacological induction of pathological R-loop accumulation to trigger catastrophic genomic instability has emerged as a mechanism-driven anti-tumor strategy. Two core approaches are pursued: direct modulation of R-loop homeostasis and synthetic lethality based on cancer-specific defects in R-loop processing (Figure 4). Collectively, these advances establish therapeutic R-loop overload as a precision oncology paradigm that selectively induces tumor-specific genomic catastrophe.

### Direct R-loop modulation

Direct targeting of R-loop regulatory factors aims to tip cellular equilibrium toward pathological R-loop accumulation, thereby inducing lethal replication stress and DNA damage in cancer cells.^85^

A major class of agents includes inhibitors of topoisomerase I and II (Topo I/II). Approved Topo I inhibitors include camptothecin (CPT) and its derivatives (irinotecan/CPT-11, topotecan/TPT, belotecan), along with the Topo II inhibitor etoposide.^104^^,^^105^^,^^106^^,^^107^ These agents induce R-loop accumulation within transcriptionally active genomic regions, including rDNA loci. Notably, proliferating cells exhibit heightened sensitivity to Topo I inhibitors (CPT and its derivatives), while quiescent cells display resistance—indicating replicative state-dependent efficacy,1^104^ spatiotemporal concordance between these DNA lesions and R-loop formation supports a model wherein R-loops drive TRC/DSB pathogenesis.^108^^,^^109^ Furthermore, TPT consistently inhibits tumor growth in organoid and non-obese diabetic (NOD)-severe combined immunodeficiency (SCID) mouse models.^110^^,^^111^

While early CPT analogs were limited by excessive toxicity, modern derivatives including CPT-11, TPT, and belotecan are approved for multiple solid tumors including non-small cell lung cancer (NSCLC), ovarian cancer, and colorectal cancer.^112^^,^^113^^,^^114^^,^^115^^,^^116^^,^^117^^,^^118^ Phase 2/3 trials reveal that combination regimens (CPT-11 + 5-FU/LV; TPT + cisplatin; belotecan + carboplatin) significantly improve objective response rates (ORR) and progression-free survival (PFS) versus monotherapy, with manageable toxicity.^119^^,^^120^^,^^121^^,^^122^^,^^123^ To overcome dose-limiting toxicities—notably myelosuppression and delayed diarrhea—next-generation agents are emerging, including SN-38 (>1,000-fold more potent than its prodrug CPT-11) and antibody-drug conjugates (e.g., hRS7-SN38). These novel compounds demonstrate breakthrough efficacy across human xenograft models of non-small cell lung, colorectal, pancreatic, and squamous cell lung carcinomas, with acceptable safety profiles in preclinical and clinical studies.^124^^,^^125^^,^^126^^,^^127^^,^^128^ Notably, sequential administration of Topo I (CPT-11/TPT) and Topo II (etoposide) inhibitors synergizes in EMT-6 breast tumor models, whereas concurrent dosing induces antagonism—highlighting temporal coordination as a critical determinant of combinatorial efficacy.^129^

Beyond topoisomerases, the DHX9 helicase inhibitor ATX968 directly destabilizes R-loop architecture. Subsequent treatment provokes sustained accumulation of RNA-DNA hybrids and exacerbates replication stress, inducing irreversible cell-cycle arrest and apoptosis in colorectal carcinoma models—an effect persisting beyond drug withdrawal.^130^^,^^131^ Its derivative ATX-559, a first-in-class clinical candidate, has secured regulatory clearance for phase 1 trials (NCT06625515), initially targeting BRCA-deficient breast cancer populations.

G4-stabilizing ligands also modulate R-loop dynamics. G4-stabilizing ligands—including pyridostatin (PDS), steroid FG, and BRACO-19—potentiate R-loop accumulation by impeding replication-transcription dynamics in human U2OS cells. This effect is markedly enhanced under BRCA2 deficiency, driving synergistic DNA lesion formation.^132^^,^^133^ BRACO-19 demonstrates single-agent antitumor efficacy: intraperitoneal (i.p.) administration induces significant tumor regression in early stage UXF1138L xenografts (including complete responses in subset cohorts) yet fails in advanced models. Oral dosing shows no activity, highlighting route-dependent bioavailability.^134^ Crucially, subtoxic BRACO-19 synergizes with paclitaxel in A431 epidermal carcinoma.^135^ Parallel evidence reveals that the clinical G4 stabilizer CX-5461 selectively kills cells via R-loop-driven cytotoxicity.^136^^,^^137^ Phase 1 trial data report a 14% partial response rate in BRCA1/2- or PALB2-mutated cancers, validating target engagement.^138^ Identification of CX-5461/HR synthetic lethality (via CRISPR screens) motivates combinatorial targeting: pairing CX-5461 with Topo I/II inhibitors to exacerbate TRC-driven R-loop accumulation, or with DHX9 inhibitors to disable R-loop resolution—dual strategies inducing catastrophic genomic instability while evading adaptive resistance.^139^

### Synthetic lethality approaches

Synthetic lethality strategies exploit cancer-specific deficiencies in DNA repair or R-loop processing to achieve highly selective tumor cell killing. In this paradigm, cancer cells with impaired R-loop resolution are sensitized to further inhibition of complementary pathways, leading to lethal R-loop overload without excessive toxicity to normal tissues.

Combinations of TOP I inhibitors and PARP inhibitors represent the most clinically advanced synthetic lethal regimen.^140^ In preclinical models of serous endometrial cancer, SN-38-driven R-loop accumulation and rucaparib-mediated PARP trapping synergistically induce lethality.^141^ Similarly, PARP inhibitor olaparib potentiates CPT-11/SN-38 in HT29/SW1116 xenografts, reducing tumor volume without severe toxicity in colon cancer.^142^ Recent study also demonstrates that olaparib and SN-38 elicit synthetic lethality in homologous recombination-proficient (HRP) ovarian cancer—overcoming intrinsic PARPi resistance in both *in vitro* HRP cell lines and *in vivo* xenografts derived from BRCA1/2-wild-type tumors.^143^ This synergy extends to glioblastoma/rhabdomyosarcoma/neuroblastoma models with combination of PARP inhibitor CEP-8983 and TOP I inhibitor CPT-11.^144^ Clinically, PARP inhibitor veliparib (ABT-888) and TOP I inhibitor TPT combination therapy exhibited acceptable safety in phase 1 (*N* = 24). Paired tumor biopsies demonstrated concomitant PAR suppression and γH2AX elevation—confirming target engagement through DNA repair inhibition and damage potentiation.^145^

Combination of TOP I inhibitors and ATR inhibitors also exert strong synthetic lethality. The novel conjugate CBX-12 (pH-sensitive TOP I inhibitor exatecan derivative) combined with ATR inhibitor ceralasertib suppresses MDA-MB-231/HCT116 xenograft progression without significant toxicity—validating its tumor-selective delivery advantage.^146^ Mechanistically, ATR inhibitors abrogate Chk1 phosphorylation, stabilizing TOP I-DNA cleavage complexes (Top1cc) to exacerbate replication stress. This synergy is conserved across models, evidenced by combinations of ATR inhibitor VE-821 and novel TOP I inhibitor LMP-400 in COLO205 colorectal and MDA-MB-231 breast carcinomas.^147^ Critically, phase 1 clinical data confirm that ATR inhibitor M6620 (VX-970) combined with TOP I inhibitor TPT induces disease stabilization at tolerable doses, with pharmacodynamic analyses revealing concomitant ATR suppression (reduced pChk1) and DNA DSBs accrual (elevated γH2AX) in tumor biopsies—providing mechanistic confirmation of target engagement.^148^

### Clinical opportunities and limitations

R-loop-targeted therapies represent a rapidly advancing frontier in precision oncology. Direct R-loop modulation and synthetic lethality strategies both induce tumor-selective genomic catastrophe by exploiting inherent defects in R-loop homeostasis. Preclinical efficacy is strong, and multiple agents have entered clinical development, including topoisomerase inhibitors, DHX9 inhibitors, G4 stabilizers, and rational combinations with PARP or ATR inhibitors. However, translating these promising strategies into routine clinical practice requires confronting a set of formidable challenges that currently temper their therapeutic impact.

Despite the promising therapeutic landscape outlined above, several critical limitations temper the clinical translation of R-loop-targeted strategies. First, tumor heterogeneity and context-dependent R-loop dynamics pose fundamental challenges, as R-loop accumulation varies markedly across cancer types, genetic backgrounds, and even within distinct tumor subclones, rendering patient stratification essential yet technically demanding. Second, on-target toxicity to normal tissues remains a major concern—particularly for agents that broadly disrupt R-loop homeostasis (e.g., topoisomerase inhibitors, DHX9 inhibitors)—given the essential physiological roles of R-loops in transcriptional regulation, immunoglobulin diversification, and chromatin organization. Third, adaptive resistance mechanisms inevitably emerge, as cancer cells can upregulate compensatory R-loop resolvases (e.g., RNase H1 and SETX), activate alternative DNA repair pathways, or acquire mutations that uncouple R-loop accumulation from replication stress. Fourth, pharmacokinetic limitations—including poor bioavailability, dose-limiting myelosuppression, and gastrointestinal toxicity—continue to constrain therapeutic windows, as evidenced by the narrow safety margins of camptothecin derivatives and the route-dependent efficacy of G4 stabilizers like BRACO-19. Finally, the lack of predictive biomarkers for patient selection and real-time monitoring of R-loop dynamics in clinical settings impedes the rational design of combination regimens and the timely assessment of therapeutic response.

Addressing these limitations will require iterative integration of mechanistic insights, advanced preclinical modeling, and biomarker-driven clinical trial designs. Nevertheless, the foundational premise remains compelling: targeting R-loops provides a powerful mechanism-based approach to precision cancer therapy. Continued development of next-generation agents and rational combination regimens will be key to unlocking the full clinical potential of R-loop-directed strategies and advancing them into routine oncology practice.

### Conclusion

R-loops function as critical integration nexuses coordinating DNA replication, transcription, and RNA processing, wherein their dynamic equilibrium exerts profound control over genomic stability and cellular fate decisions. Dysregulated R-loop homeostasis is increasingly recognized as both a catalyst and perpetuating mechanism driving oncogenesis across multiple malignancies. The observed tumor-type-specific R-loop landscapes necessitate the development of integrated multi-omics frameworks to enable precision targeting of R-loop vulnerabilities. Advancing this paradigm will require concerted interdisciplinary integration to fully exploit the therapeutic potential inherent in R-loop biology.

## Acknowledgments

This work was supported by the 10.13039/501100001809National Natural Science Foundation of China (grant 32270777to Z.T.); the Independent Science and Technology Innovation fund of 10.13039/501100007925Huazhong Agricultural University (grant 2662024SYPY001 to Z.T.), the 10.13039/501100001809National Natural Science Foundation of China (grant number 82103260 to K.N.); the 10.13039/501100013105Shanghai Rising-Star Program (grant number 22QA1407100 to K.N.); the Excellent Youth Cultivation Program of Shanghai Sixth People’s Hospital (grant number ynyq202204 to K.N.) and the Fundamental Research Funds of Shanghai Sixth People’s Hospital (grant number X-2490 to K.N.).

## Author contributions

All authors contributed to the writing of this article.

## Declaration of interests

The authors declare no competing interests.
